# Modern models for predicting bankruptcy to detect early signals of business failure: Evidence from Montenegro

**DOI:** 10.1371/journal.pone.0303793

**Published:** 2024-05-21

**Authors:** Milica Vukčević, Milan Lakićević, Boban Melović, Tamara Backović, Branislav Dudić

**Affiliations:** 1 Faculty of Economics Podgorica, University of Montenegro, Podgorica, Montenegro; 2 Faculty of Management, Comenius University Bratislava, Bratislava, Slovak Republic; University of Barcelona: Universitat de Barcelona, SPAIN

## Abstract

This paper explores predicting early signals of business failure using modern models for bankruptcy prediction. It reviews how continuous operations enhance market value, strengthening competitiveness and reputation among stakeholders. The study involves medium and large companies in the Montenegrin market from 2015 to 2020, comprising 30 bankrupt and 70 financially stable firms. Logistic regression is also employed to create a logit model for early detection of bankruptcy signals in companies. This research establishes the empirical validity of modern models in predicting business failure in the Montenegrin market, particularly through logistic regression. Significant indicators, such as the Degree of Indebtedness (DI) and turnover ratio of business assets (TR), exhibit strong predictive power with a p-value less than 0.001 according to Likelihood ratio tests. The paper underscores the potential benefits of bankruptcy prediction for both internal and external stakeholders, especially investors, in enhancing the competitiveness of Montenegro’s large and medium-sized companies. Notably, the research contributes by bridging the gap between theory and practice in Montenegro, as bankruptcy prediction models have not been extensively applied in the market. The authors suggest the possible applicability of the created logit model to neighboring countries with similar economic development levels. In that sense, the concept of predicting bankruptcy is positioned as integral to corporate strategy, impacting the overall reduction of bankruptcies. The paper concludes by highlighting its role as a foundation for future research, addressing the literature gap in the application of bankruptcy prediction models in Montenegro. The created logit model, tailored to the specific needs of Montenegrin companies, is presented as an original contribution, emphasizing its potential to strengthen the competitiveness of companies in the market.

## Introduction

A dynamic and unstable business environment, the growth of competition, pronounced changes, and specific operations in crisis conditions are just some of the factors that characterize the way companies operate on the market nowadays [1–3]. Therefore, an important question for stakeholders is whether the company, due to numerous market challenges, will operate in the future or will go bankrupt [4,5]. The above points to the need to predict the bankruptcy of the company [6], so the developed models are based on indicators from financial reports, which allow predicting the success of the business entity in the future [7]. The importance of the indicators from the financial report is shown by the research of the author Rodríguez-Valencia [8], which used selected indicators to estimate the value of the company. Users of financial statements want to predict the future performance of companies [9,10] to make adequate business decisions, from which they will realize a certain benefit in the future and have the highest return on investment. Additionally, the requirements of the International Financial Reporting Standards and the International Auditing Standards indicate the issue of the responsibility of the company’s management, as well as the independent audit of the financial statements, so that the assumption of the continuity of the company’s operations is sustainable [11–13]. The previously stated, as well as the fact that the models for early detection of bankruptcy of companies are gaining more and more importance, became the motive for conducting this research. In this regard, an increasing number of bankruptcy proceedings is evident, which is also confirmed by the Eurostat report [14]. In this way, early indicators of business failure can be observed in time, so that measures can be taken by the management to improve the company’s business. To achieve this, decision-makers should have adequate knowledge, especially in the field of finance and accounting. That way, based on information obtained from a set of financial reports, the management would be able to recognize early signals of business failure. Forecasting the business failure of companies, according to previous research, can be done using a traditional model (based on selected indicators) [15–17], but also by using existing, modern models (Altman Z“model, Springate model, Zmijewski model, Kralicek’s model, BEX model, logit model) [18–22] to recognize early signals of business failure, i.e. determine whether the analyzed company will go bankrupt or not.

However, this topic is still insufficiently researched in countries with a low and medium level of development, including Montenegro, which is one of the reasons for conducting this research. The previous research was mainly based on the application of the model to a sample of small and medium-sized enterprises. In contrast to such research, this work will be based on the application of a model for predicting business failure on the example of large and medium-sized enterprises. It is important to point out that by analyzing the literature, according to the author’s knowledge, there are very few papers that include the integral application of several models for predicting business failure. It is important to find out which models can be considered reliable for application, all to prevent the trend of increasing the number of bankruptcy proceedings, which is also evident in Montenegro. The aforementioned, along with the fact that, according to the author’s knowledge, no comprehensive research in this field has been carried out in Montenegro yet, represents an observed literal gap. Analyzing the application of the model for predicting business failure would also ensure compliance with the principle of going concern as one of the most important concepts for business operations as well as preparing financial statements [23], which is an additional motive for conducting this research.

In this way, the research will provide insight into whether it is necessary to create a new model, adapted to the Montenegrin market, and whether the created logit model will enable decision-makers to predict the bankruptcy of a company based on pre-selected indicators, or whether the existing models have a satisfactory level of reliability. In this way, a clearer picture is provided to the decision-makers regarding the operations of the company. It also indicates the measures that the management should take to improve the business of the company, and therefore its competitiveness in the market. In this way, the probability of companies achieving business failure would be reduced.

The paper consists of four parts and, in addition to the introduction, contains an overview of previous research, a description of the methodology, research results and discussion, conclusion, and implications, as well as limitations and recommendations for future research.

## Literature review

Rapid changes in business, especially during crises and an unstable environment with advanced technology and increased competition, significantly affect the company’s operations. Decision makers must continuously research to make more confident business decisions. In other words, decision-makers, due to the increasing number of business failures of companies on the market [14], should analyze financial reports. Financial report analysis serves as a tool for applying bankruptcy prediction models, utilizing various financial indicators to assess a company’s performance compared to the previous year. These indicators identify areas for improvement, helping prevent company bankruptcy. Business failure occurs when a company, regardless of its size or activity, cannot meet its obligations. Therefore, companies that have experienced business failure have certain indicators in the form of financial indicators, which point to unsatisfactory ways of doing business operations [24]. For that reason, models for the early detection of company bankruptcy, created based on financial indicators, are gaining more and more importance, so it is not surprising that this issue is the subject of research by numerous authors. Thus, financial distress is one of the most important factors affecting the solvency of companies. It has influenced the development of models for assessing financial problems in industrial and financial sectors [25].

In general, models for predicting company bankruptcy can be divided into three groups. The first group consists of models based on statistical methods. These are univariate and multivariate models. This group involves MDA, logit, and probit models [5]. The second group consists of artificial intelligence models, which are more complex to apply. Within these models, there are Decision Trees, Neural Networks (multilayer perceptron and the novel quantum neural network), and Support Vector Machines (SVM) [26–29]. The third group consists of theoretical models for which statistical analysis supports theoretical foundations [20,30]. It is important to emphasize that Dynamic Stochastic General Equilibrium (DSGE) and Vector Autoregressive (VAR) models are becoming more and more important and relevant in analyzing business cycles and detecting recessions [31]. However, models for predicting company bankruptcy can be divided into traditional and modern models [32] and the authors note that traditional models involve indicators related to property, financial, and profitability analysis based on financial statements. In contrast, modern models involve the Altman model, Springate model, Zmijewski model, BEX model, and others. Until the appearance of Beaver’s research, the first study dedicated to business failure was developed by Charles L. Merwin, and it was considered the most significant study until the 1960s. However, the univariate analysis of Beaver [33], who based his study on the sample of 79 bankrupt companies, will be of great importance for the development of multivariate models for predicting corporate bankruptcy. The first multivariate model was developed by Altman in 1968, based on five analytical indicators, and was originally developed for the needs of the American market. According to this model, if the company had a Z-score value greater than 3, the company was characterized by financial stability. However, since Altman’s original model only applied to manufacturing companies listed on the stock exchange, over time there was a need to develop models that also apply to other economic activities, regardless of whether the company is listed on the stock exchange or not. Thus, the Z’ score model was developed for companies that are not listed on the stock exchange, and the Z" score model, was intended for companies that do not belong to the production sector Altman et al. [34].

In addition to Altman’s model, the Springate model is also important for predicting company bankruptcy, which classifies companies into two groups: healthy companies and companies with a high probability of bankruptcy in the next year. The model was formed based on four indicators (Assets to working capital, EBIT / total assets, EBT / current liabilities, and Total assets/sales) [35] with a threshold value of 0.862 [36]. An important model for predicting company bankruptcy was developed by Ohlson in 1980, forming a logit model, to overcome the shortcomings of the discrimination function [37]. Ohlson criticizes previous research on predicting business failure for using paired samples and not meeting the mandatory assumptions of discriminant analysis. Therefore, logistic regression does not require normality of the distribution of independent variables, which is a commitment in application to the discriminant function [38]. Also, the Zmijewski model is well-known in the literature, based on three analytical indicators (Net Income/Total Assets, Total Liabilities /Total Assets, and Current Assets / Current Liabilities) [39] and gives the value of the probability function between 0 and 1. The probability of a company going bankrupt within the next year increases as the value approaches 1[40].

Kralicek’s DF indicator is also used for identifying a crisis in a company for European companies. This indicator can record positive and negative values, where a positive value indicates the company’s solvency, while a negative value indicates insolvency. The value of this indicator ranges from -1 to 3. Companies with a DF indicator value greater than 3 belong to companies with excellent financial stability [41]. The IN99 model was developed in the Czech Republic based on the application of four analytical indicators (assets/liabilities; EBIT/assets; revenue/assets; assets/(short-term liabilities and short-term bank loans)) to assess the success of the company’s operations by the owner [41]. It indicates that if the value of the indicator is more than 2.07, it is a healthy company, while if the value is less than 0.684, the company is bankrupt. The indicator value, obtained between the upper and lower limits, indicates that the company is in the "grey" zone [42]. In contrast to the IN99 model, the IN05 model was developed in the Czech Republic for the needs of creditors to evaluate the company’s performance. Depending on the value of the indicator, companies are classified as healthy ones, if the value is more than 0.5, that is, the company is bankrupt if the value is less than 0.9. In addition to the previously mentioned models, the BEX model is developed for the Croatian market and intended for evaluating the performance of companies on the capital market. It was formed based on four indicators (the ratio between (EBIT) and total assets, the ratio of net operating profit to equity capital, the ratio of working capital to total assets, ratio of theoretically free money from all activities) [43] from the financial report and it indicates that if the value of the BEX indicator is greater than 6, the company operates with outstanding results, while if the value is less than 0, the company operates on the unsatisfactory way [44]. The application of neural network models, decision trees Support Vector Machines, and Restricted Boltzmann Machine, represents new approaches to predicting bankruptcy based on artificial intelligence [28]. The previously mentioned models of artificial intelligence can find a foothold in the market, but they are complex for application and interpretation of the obtained results. However, these models still do not always provide the most accurate results [20]. How important statistical models are for the analysis of this issue for decision-makers is best shown by the research of numerous authors, who apply them to examine whether there is a probability that the analyzed company records a business failure on the market [24,45,46]. Unlike the previous authors, the logit model and Altman’s Z score were applied to predict the bankruptcy of small and large companies in Asia, whereby the subject of the analysis is to determine which of these two models is more reliable for predicting business failure [47]. A similar study was conducted in Jordan where Altman’s Z score and the Kida model were applied to predict corporate bankruptcy [48]. The research conducted in Pakistan examined the ability of financial indicators and Altman’s Z model to predict bankruptcy in the textile sector. [16]. In the same country, a study was conducted on the application of the Altman and Abbas model to predict bankruptcy of non-financial companies listed on the stock exchange [49].

The application of Altman’s Z score is shown in research conducted in Sri Lanka, where the subject of research was the possibility of predicting the bankruptcy of listed manufacturing companies [18]. Unlike the previously mentioned, the application of Altman’s model in the market of the same country was analyzed, but in this case for the trade sector [50]. The prediction of company bankruptcy was also carried out in Tehran, to determine the possibility of predicting bankruptcy for listed companies using the Altman, Springate, Zmijewski, and Grover models [21]. The results obtained using these models indicated good predictive capabilities for predicting business failure. The application of artificial intelligence models was the subject of research to compare the ability to predict the bankruptcy of companies using these models to Altman’s Z model [51]. Unlike the previous authors, who aimed to analyze the application of the model for predicting the bankruptcy of companies in one country for a specific market segment, the author Alaminos et al. [20], applying the logit model, investigated the possibility of forming a global model that can be applied to predict the bankruptcy of companies. Thus, it covered the regions of Asia, Europe, and America. Logistic regression was also applied in Albania to predict the bankruptcy of companies [52]. In order to predict the bankruptcy of small companies in Romania, during the financial crisis, the models of Altman, Toffler’s model and the logistics model were applied [53]. Logit and probit models were used to analyze the potential for predicting company bankruptcy in the Russian market [54]. The application of Altman’s model was also tested in Italy, on companies listed on the stock exchange [55].

The possibility of applying a model for predicting company bankruptcy was implemented in Croatia analyzing company bankruptcy using logistic regression, on the example of small and medium-sized enterprises [56]. Also, in the same country it was examined the possibility of predicting company bankruptcy, based on relevant financial indicators of liquidity, solvency, and profitability for small and medium enterprises [57].

By applying the corrected model Z", an analysis of the creditworthiness of companies in Serbia was carried out to determine the probability of bankruptcy [58]. In the Serbian market, it was researched the example of manufacturing companies, using logistic regression, to analyze the possibility of predicting company bankruptcy [59]. The beginning of research on models for predicting company bankruptcy in Montenegro was related on the application of seven selected financial indicators and four models for the possibility of predicting the bankruptcy of a company [32].

Based on previous research and the variety of models and methods available in the field of business difficulties research, it can be concluded that the most widely used models in the markets are those based on statistical methods and financial indicators [60,61]. Also, it can be seen that this problem is current and popular among authors around the world and models for predicting bankruptcy are applied regardless of the size, activity of the company, and the market in which they operate. However, despite the great importance of these models for maintaining the stability of the company’s operations, they are rarely applied in Montenegro, which is also an observed literature gap. Therefore, this paper will analyze the possibility of applying modern models for the early detection of bankruptcy of companies in the Montenegrin market. The goal is to determine the reliability of their application for the mentioned market and obtain the parameters of financial analysis that contribute the most to predicting business failure, using logistic regression, intended for the Montenegrin market and economy, to ultimately reduce the number of bankruptcy proceedings. In this way, the gap between theory and practice will be bridged and a conclusion will be reached on whether it is necessary to create a new model for the Montenegrin market or whether the existing models have nevertheless, a satisfactory level of reliability for the application. It should make it easier for managers to make business decisions. It is important to point out that maintaining a long-term business company’s competitiveness in the market is significant, considering that Montenegro needs to have a stable economy. However, to achieve this goal, healthy large and medium-sized companies are necessary, especially those in which the state has a majority share. The aforementioned is also significant due to the fulfillment of the conditions for accession to the European Union, specifically Chapter 6, which is related to corporate governance.

## Materials and methods

Taking into account previous research, as well as the identified literature gap and the aim of the research, this paper will examine whether modern models such as Altman’s Z" model, Springate model, Kralicek’s model, Zmijewski model and BEX model [21,32,34,36,40,41,57] reliable for application on the Montenegrin market. Previous research has focused on the analysis of individual models, but until now, according to the author’s knowledge, no research has been conducted that had a combination of models for predicting business failure as a subject, on the example of large and medium-sized companies. Following the above, the hypotheses were formulated:

*H1*: *Altman’s Z”Model is reliable for predicting the business failure of large and medium-sized enterprises in Montenegro*.
*H2*: *The Springate model is reliable for predicting the business failure of large and medium-sized enterprises in Montenegro*.
*H3*: *The Zmijewski model is reliable for predicting the business failure of large and medium-sized enterprises in Montenegro*.
*H4*: *Kralicek’s DF model is reliable for predicting the business failure of large and medium-sized enterprises in Montenegro*.
*H5*: *The BEX model is reliable for predicting the business failure of large and medium-sized enterprises in Montenegro*.

Global and regional research used frequency-based indicators from previous studies to create a logit model for predicting business failure [24,45,52]. However, despite this, this model was not the subject of the author’s analysis and application in this area in Montenegro, which will further overcome the observed literature gap. Therefore, the sixth hypothesis was formalized and will be tested using logistic regression. In other words, by applying logistic regression, it will be determined which of the analyzed indicators of financial analysis (degree of indebtedness, business asset turnover ratio, EBIT, general liquidity ratio, ROA, and ROE), selected based on previous research) have the greatest statistical significance for predicting business failure of large and medium enterprises in Montenegro.

*H6*: *The degree of indebtedness and the turnover ratio of business assets have a statistically significant influence on the prediction of business failure of enterprises in Montenegro*.

**Fig 1:**
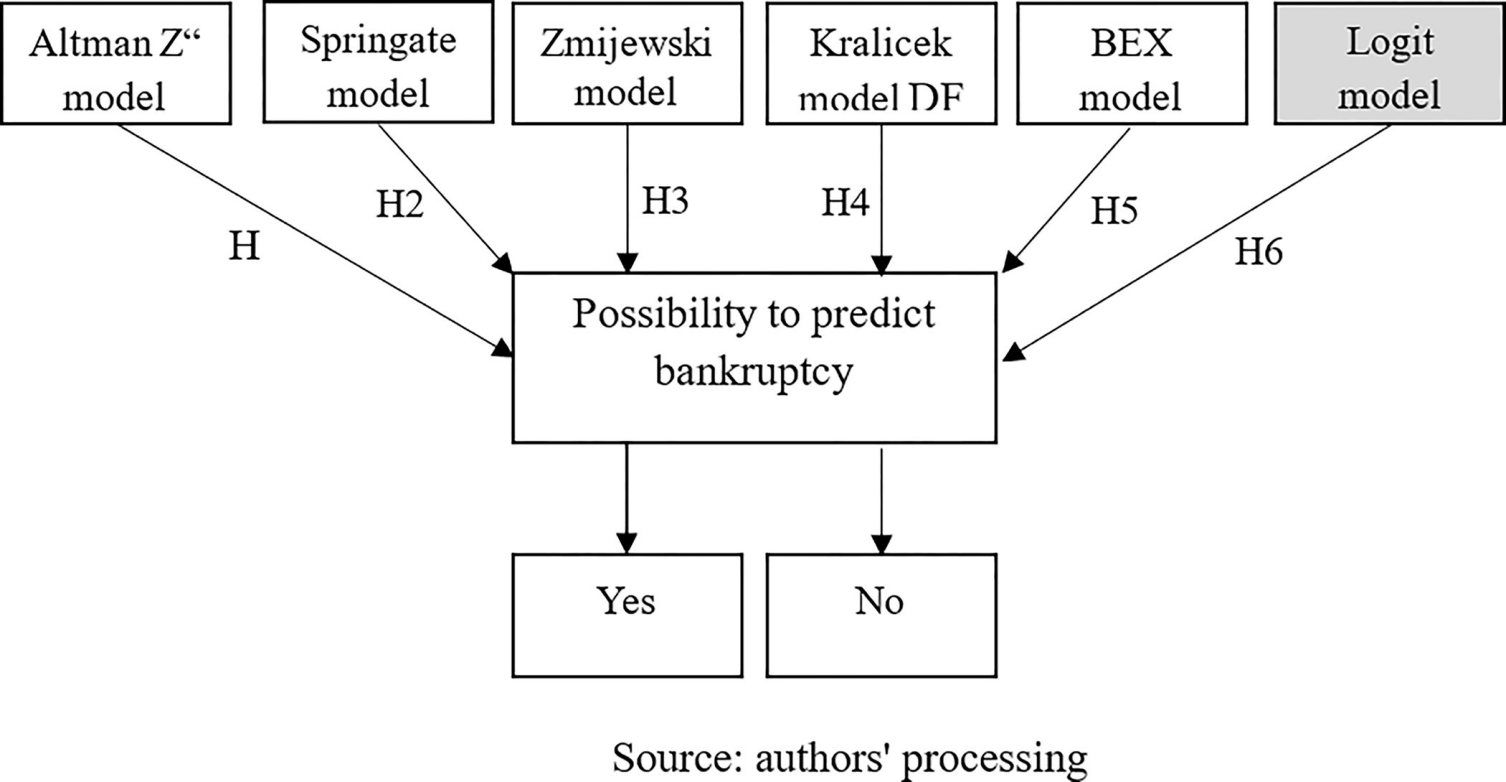
Conceptual model of research.

Following the previously formulated hypotheses, a conceptual research model was created, shown in Fig 1.

Based on the conceptual model, it can be seen that the research is based on the application of five moderns, and specially created logit model intended for the Montenegrin market. For each of the five models, data will first be collected based on the official financial reports of the company (Balance Sheet, Income Statement, and Cash Flow Statement) and then the indicators will be calculated. Each of these models has its score, and according to the value obtained from the analysis, it will be determined whether the company operates in the bankruptcy zone, the gray zone of business with the possibility of recovery and improvement, or whether it is still a question of financially healthy companies. As a special type of modern model, logistic regression will be applied in the paper to create a logit model, created based on the specifics of the Montenegrin economic environment.

The methodology of this research is based on the application of modern models (Altman’s Z" model, Springate model, Kralicek’s DF model, Zmijewski’s model, and BEX model) to detect early signals of the possible bankruptcy of the company. It is important to point out that the models were selected based on their popularity, relevant literature, availability of data, as well as technological possibilities for conducting research. The research involved medium and large companies that operate, or operate in the Montenegrin market. In other words, according to the Law on Bankruptcy and the list obtained by the Central Registry of Montenegro, companies that went bankrupt in the period from 2015 to 2020 were selected. Therefore, medium and large companies that went bankrupt were included in the research. Besides that, more than twice as many healthy companies will be included in the research, which is in line with previous studies [24,52,62]. The criterion for selection of large and medium-sized enterprises was based on the Law on Accounting, which defined medium-sized enterprises as those with an average number of employees in the business year up to 250, total annual income up to 40,000,000 €, or total assets up to 20,000.000 €. Large companies are those that meet two of the three criteria IFAC [63]. These categories of business entities are important because they employ the largest number of employees in their companies, viewed per individual company, and generate the highest business results, which is particularly important for the state and due to the establishment of an adequate fiscal policy. In addition, these companies must compile and publicly publish a complete set of financial reports, which is important due to the availability of data, and therefore the application of models for predicting business failure. All of the above is the basis for the establishment of good corporate practice in Montenegro, and the company’s operations on a healthy and sustainable basis. It means that the application of a model for predicting business failure is a segment that should be analyzed in companies to fulfill the principle of going concern and enable good corporate governance. This is especially important taking into account that Montenegro is in the process of joining the EU and the important chapter that it needs to close is related to the arrangement of the corporate governance and the establishment of the foundations that will greatly contribute to that goal. It is important to emphasize that in some large and medium-sized enterprises in Montenegro, the state is the majority owner, and therefore the operations of these companies are particularly important for the Montenegrin market and the economy as a whole.

For each observed company (a sample of 30 companies) that went bankrupt, modern models will be applied to assess the reliability of their application for predicting bankruptcy. For example, if the company went bankrupt in 2016, the application of modern models will be carried out based on the financial statements for 2015, to see whether these models predict the bankruptcy of the company in the following year, or this case in 2016. The final decision on whether the models are reliable or not is made based on a comparison of the number of bankrupt companies and the number of bankrupt companies. For healthy companies, the research was conducted on a sample of 70 large and medium-sized companies, based on financial reports (balance sheet and income statement), taken from the website of the Revenue and Customs Administration of Montenegro [64]. It is important to emphasize that this segment of the research was conducted in such a way that the data was taken from the white list of taxpayers for 2020, and based on the report from 2019, we want to predict whether the company will record business continuity in 2020. After the application of modern analysis, with the help of logistic regression, based on the most represented financial indicators in the literature (Debt ratio, Asset Turnover Ratio, EBIT, Current Liquidity Ratio, ROA, and ROE) it is possible to make the conclusion which parameters have the most influence on predicting the bankruptcy of large and medium-sized enterprises in Montenegro.

Modern models that will be applied in this research are Altman’s Z” model, the Springate model, the Zmijewski model, Kralicek’s model, the BEX model, and Logistic regression. It is important to point out that each of the mentioned models was explained in the previous part of the paper through a review of the literature. In this part, the equations of the model will be given with the analytical parameters that make them up, as well as the threshold values based on which the decision on the financial condition of the company is made. The foregoing is given in Table 1.

**Table 1:** Overview of modern models for predicting business failure.

Models	Equation	Indicators	Threshold values	Source
**Altman’s Z”model**	*Z*" = 6.56·X1+3.26·X2 +6.72·X3+1.05·X4	X_1_—Working Capital/Total Assets X_2_—Retained Earnings/ Total Assets X_3_—EBIT/ Total Assets X_4_—Book value of equity/Book value of total liabilities	Z" > 2.6—financially successful companies Z" > 1.11, and Z" <2.59, the gray area of business operations, Z" < 1.10—financially unsuccessful companies	[58], [65], [66]
**Springate model**	SS = 1.03·X1+3.0·X2 +0.66·X3+0.4·X4	X_1_—Working Capital/Total Assets X_2_—Net profit before interest and tax / Total assets X_3_—Net profit before tax / current liabilities X4—Sales / Total assets	SS < 0.862, Potentially Bankrupt.	[66], [67], [68]
**Zmijewski model**	ZZ = −4.336−4.513·X1 +5.679·X2+0.004·X3	X_1_—Net Income / Total Assets X2—Total Liabilities / Total Assets X_3_—Current Assets / Current Liabilities	A probability function greater than 0.5 represents a higher probability of bankruptcy.	[36], [67], [69]
**Kralicek’s model**	DF = 1.5·X1+0.08·X2 +10·X3+5·X4+ 0.3·X5+0.1·X6	X_1_—net cash flow/total liabilities X_2_—total assets/total liabilities X_3_—profit/total assets X_4_—profit/operating income X_5_—inventories/operating income X_6_—operating income/total assets	DF >3.0 Excellent DF >2.2 Very good DF >1.5 Good DF >1.0 Average DF>0.3 Bad DF = 0.3 Beginning of insolvency DF≤0.0 Moderate insolvency DF≤-1.0 The striking insolvency	[41], [70]
**BEX model**	*BEX* = 0.388·EX1+0.579·EX2 +0.153·EX3+ 0.316·EX4	EX_1_—EBIT/ Total Assets EX_2_—Net Income /(Equity · cost of capital) EX_3_—Working Capital / Total Assets EX_4_ - (5·(Net income + depreciation + amortization))/Total liabilities	Greater than 6.01 for 4 years in a row—World class Greater than 6.01- World-class candidate 4.01–6.00 Excellent 2.01–4.00 Very good 1.01–2.00 Good 0.00–1.00 Borderline Negative—The company’s existence threatened	[44]
**Logistic regression**	$P(Z)=\frac{1}{1+exp(-Z)}=1/(1+exp(-(a+b1x1+\cdots +bnXn))$	P_(Z)_˗the probability of an event, exp ˗ base of natural logarithm, xi—(i = 1,. . .,n) ˗˗ independent variable, α˗ line segment bi-(i = 1,. . .,n)–regression coefficients.	The probability ranges from 0 to 1	[20], [71]

Source: Authors’ processing.

## Results and discussion

To examine the claims presented through the formulated hypotheses, modern models were applied. Table 2 shows how many companies for which the applied models predict bankruptcy. Also, it represents how many of them would continue to operate according to the going concern principle.

**Table 2:** Modern models–companies in bankruptcy.

Models	Results		%
**Altman Z” model**	Bankrupt	12	0.40
	Non—Bankrupt	18	0.60
**Springate model**	Bankrupt	30	1.00
**Zmijewski model**	Bankrupt	24	0.80
	Non—Bankrupt	6	0.20
**Kralicek model**	Bankrupt	30	1.00
**Bex model**	Bankrupt	30	1.00

Source: Authors’ processing.

The previous table shows that most of the applied models, out of a total of 30 large and medium-sized companies that are bankrupt, predicted that the observed companies would go bankrupt. However, the analysis found that Altman’s Z" model only predicted bankruptcy for 40% of the companies in the sample, which was not expected, especially considering that it is a model that records good predictive performance in most of the studies in which it was applied. However, although this model is intended for developing markets, the results of the research showed that it is not reliable enough for application in the Montenegrin market, thus the claim given by the first hypothesis was not confirmed. The result obtained in this way follows the findings obtained in the research of the author Heaton [72]. Evident differences in the degree of reliability of the application of Altman’s Z" in economies, such as the Montenegrin one, stem from the different economic environments and legal regulations that characterize the operations of companies.

Unlike Altman’s Z" model, Zmijewski’s model classified 24 companies, or 80% of them, as bankrupt, which indicates that it can be applied to the Montenegrin market to predict business failure. In this way, the third hypothesis was confirmed, and it can be considered reliable for application in Montenegro. That the Zmijewski model is significant for predicting business failure was also confirmed through research by Aminian et al. [21]. The aforementioned finding is particularly important because it was conducted on a sample of large companies, which proved that the Zmijewski model has a foothold for predicting bankruptcy over defined business entities. Also, that it is a model that can be considered reliable, was confirmed in the research conducted in Serbia, which has a similar level of development as Montenegro Index of Global Competitiveness [73]. Significantly, the Springate, DF, and BEX models predict that all analyzed companies (which are bankrupt) will indeed go bankrupt, which is a 100% effect, thus confirming the second, fourth, and fifth hypotheses. That the Springate model is reliable for predicting business failure was also confirmed through the results of research by Wahyuningsih & Venusita [74] and Aminian et al. [21]. Unlike the author Salkić [75], who indicated in his research that Kralicek’s DF model cannot be considered reliable for predicting the bankruptcy of companies in Bosnia and Herzegovina, which is also a developing country like Montenegro, the findings of this research indicate 100% reliability on a defined sample. A significantly lower level of reliability was also pointed out in the research of the author Mijić [76], who indicated that by applying Kralicek’s DF model, bankruptcy is predicted for 60% (observed from the point of view of those who are bankrupt). When it comes to the results of the BEX model, its reliability on the Montenegrin market is almost the same as the market for which it was created [44]. Additionally, to show whether these models predict the continuity of business of healthy companies, modern models were also applied, and the results are shown in Table 3.

**Table 3:** Modern models for predicting bankruptcy—"healthy companies".

Models	Results		Probability
**Altman Z” model**	Bankrupt	1	0.01
	Non—Bankrupt	69	0.99
**Springate model**	Bankrupt	36	0.51
	Non—Bankrupt	34	0.49
**Zmijewski model**	Bankrupt	11	0.16
	Non—Bankrupt	59	0.84
**Kralicek model**	Bankrupt	20	0.29
	Non—Bankrupt	50	0.71
**BEX model**	Bankrupt	5	0.07
	Non—Bankrupt	65	0.93

Source: Authors’ processing.

Based on the previous table, it can be seen that the most reliable model that indicates that companies will continue to operate in the next year is Altman’s Z" model, which for 69 companies, i.e., 99% of the sample, indicated the continuation of operations. In addition to Altman’s model, the BEX model also recorded a level of reliability higher than 90%, which indicated business continuity for 65 companies in the following year. In addition to these two models, the Zmijewski and DF models also achieve significant reliability, which classified 84% and 71% of companies, respectively, as operating following the principle of going concern. The model that is considered insufficiently reliable is the Springate model, which characterizes only 34 companies as healthy. Therefore, it can be concluded that all the analyzed modern models, except for the Springate model, can be applied in the Montenegrin market to assess the continuity of business of the analyzed companies.

The results of the logistic regression show that by applying the reliability quotient test to examine the significance of the coefficients in the model, it was indicated that the values of the credibility quotient test are statistically significant, which means that the model with introduced financial indicators is statistically significant (Table A1 in S1 Appendix).

The results of the Likelihood Ratio test indicate which one of the listed financial indicators has a statistically significant effect on explaining the probability of bankruptcy of a certain company. The importance of this test is reflected in the fact that the significance of the influence of variables is seen through the measurement of individual independent influences on the dependent variable, which cannot be done in the case of evaluating logistic regression. The results of the Likelihood ratio test indicate that for predicting the bankruptcy of the company, the influence of the financial indicators Degree of Indebtedness (DI) and the turnover ratio of business assets (TR) is significant. Table A2 in S1 Appendix provides an assessment of the parameters included in the creation of the logit model with a confidence interval of 95%

To assess the significance of each financial indicator to assess the company’s bankruptcy, it is necessary to analyze the results of the Wald test. The initial hypothesis of the Wald test claims that the value of the parameter with the given explanatory variable is equal to zero, that is, that variable does not significantly affect the assessment of company bankruptcy. The results of the Wald test for the initial logistic regression are identical to the results of the reliability coefficient test and indicate that DI and TR are the only financial indicators that can statistically significantly influence the assessment of company bankruptcy. Therefore, a new logistic regression of company bankruptcy assessment was evaluated below, in which the financial indicators DI and TR appear as explanatory variables (Tables 4–6).

**Table 4:** Case processing summary.

Case Processing Summary			
		N	Marginal Percentage
Bankruptcy	.00	70	70.0%
	1.00	30	30.0%
Valid		100	100.0%
Missing		0	
Total		100	
Subpopulation		100^a^	
a. The dependent variable has only one value observed in 100 (100.0%) subpopulations.			

Source: Authors’ processing.

**Table 5:** Model fitting information.

Model Fitting Information				
Model	Model Fitting Criteria	Likelihood Ratio Tests		
	-2 Log Likelihood	Chi-Square	df	Sig.
**Intercept Only**	122.173			
**Final**	59.742	62.430	2	< .001

Source: Authors’ processing.

**Table 6:** Likelihood ratio tests.

Effect	Model Fitting Criteria	Likelihood Ratio Tests		
	-2 Log Likelihood of Reduced Model	Chi-Square	df	Sig.
Intercept	72.144	12.401	1	< .001
DI	97.304	37.561	1	< .001
TR	95.932	36.189	1	< .001

Source: Authors’ processing.

The results of the reliability coefficient test showed that the bankruptcy model with explanatory variables DI and TR are more significant than the bankruptcy model without these explanatory variables.

The chi-square statistic is the difference in -2 log-likelihoods between the final model and a reduced model. The reduced model is formed by omitting an effect from the final model. The null hypothesis is that all parameters of that effect are 0.

The coefficient ratio test, which examines the individual influence of the explanatory variables of the new logistic regression model of bankruptcy, shows that DI and TR are significant variables for assessing the chance of bankruptcy of a certain company, which was confirmed by parameter estimation with a confidence interval of 95% (Table 7).

**Table 7:** Parameter estimates.

Bankruptcy ^a^		B	Std. Error	Wald	df	Sig.	Exp(B)	95% Confidence Interval for Exp(B)	
								Lower Bound	Upper Bound
.00	Intercept	1.916	.640	8.954	1	.003			
	DI	-.050	.012	16.248	1	< .001	.951	.929	.975
	TR	3.082	.869	12.593	1	< .001	21.805	3.974	119.628

Source: Authors’ processing.

a. The reference category is non—bankruptcy.

The initial hypothesis of the Wald test, according to which the parameters along with the explanatory variables DI and TR are equal to zero, can be rejected with a risk of error of 1%. It can be concluded that all parameters of the logistic regression of the assessment of company bankruptcy are statistically significant. Companies that did not go bankrupt have a 0.049 times lower chance of having a higher level of indebtedness than companies that went bankrupt. Companies that are not currently in a state of bankruptcy have a 21,085 times higher turnover ratio of business assets compared to companies that have gone bankrupt. The importance of the TR parameter was also confirmed in the research of the author Ptak-Chmielewska [77], who, unlike this research, applied LDA and SVM methods and found that the turnover ratio of business assets is significant for predicting the bankruptcy of companies. The authors Ogashi et al. [78] used logistic regression to prove that the turnover coefficient of business assets and the degree of indebtedness are statistically significant variables for predicting business failure, among others. This finding is in contrast to the study conducted by Ptak-Chmielewska [77].

## Conclusions

Bearing in mind presented in this paper, it can be concluded that early signals of business failure of companies in Montenegro can be observed based on financial reports. However, for this to be possible, the financial reports must be prepared following IAS, IFRS, and EU Directives, while it is important to emphasize that the accounting information contained in the reports should be relevant and precise, thus reducing the possibility of materially significant errors, which greatly influence decision makers when making business decisions.

In this sense, the quality of financial reports should be confirmed by independent persons, i.e., auditors, so an additional goal is to increase the quality of the audit of financial statements. In this way, it is possible to influence the provision of financial stability, which is increasingly threatened by the presence of various forms of speculation, which in the long term may threaten business continuity [79]. This is because the financial failure of companies represents a problem for the economy of a country, and therefore it is not surprising that bankruptcy processes have become an international problem, due to the internationalization of business. Based on the above, it is clear why the number of bankruptcy proceedings is seen as a kind of indicator of the strength and success of national and regional economies, and therefore it is important to have accurate and reliable instruments based on adequate accounting information, for early bankruptcy prediction.

Many bankruptcy prediction models have been developed in theory and practice. In addition to the traditional model, based on the analysis of the property, financial, and profitability position of the company, in practice, there are models based on mathematical and statistical methods. However, to apply modern models, it is necessary to know and understand the indicators of financial analysis, as the basis for the analysis of the traditional model. This is because some modern models were created by combining financial indicators.

The results of modern models applied in the Montenegrin market depend on the model that was applied and the threshold value about which it was observed whether the company achieves good or bad business operations. Altman’s Z" model classified 40% of the 30 analyzed companies in bankruptcy as going bankrupt, while the remaining 60% indicated that they would continue doing business in the future. Analysis shows that out of the 70 analyzed "healthy" companies, modern models 69 entities classify as healthy ones, and only one company, according to the results, would not continue to operate in line with the going concern principle. Unlike Altman’s Z" model, the Springate model predicted for all bankrupt companies that it will start, which makes the effect of this model 100% for the sample of bankrupt companies, while the analysis of "healthy" companies shows that this model, out of a total of 70 companies, predicted business continuity only for 33. Furthermore, the Zmijewski model showed that out of 30 bankrupt companies, 80% of them will go bankrupt, while out of a sample of 70 healthy companies, it classified 60 as companies with good business performance. Kralicek’s and BEX’s models, like European models, predict bankruptcy for all companies that are in bankruptcy, while for "healthy" companies, Kralicek’s model predicts that 75% of them will continue to operate in the next year. The BEX model indicates that out of 70 companies, only 11 are doing well, while 4 companies are classified as going bankrupt. This model showed that as many as 55 companies are characterized by borderline operations, which means that their business excellence is positive, but not satisfactory.

Therefore, a general conclusion can be made that all but Altman’s Z" model can be said to have a sufficient level of reliability for predicting early signals of bad business. Also, by applying logistic regression, it was determined that the degree of indebtedness of the company and the turnover ratio of business assets have the greatest influence on predicting the bankruptcy of companies in Montenegro.

It is important to point out that the research results emphasized the unsatisfactory operations of financially "healthy" companies, which should become a clear signal to the decision-makers in the company. This is because if early signals are noticed in time, timely action can be taken and bad business can be prevented in the future. On the aforementioned, the practical, theoretical, and methodological contribution of the research is based.

The main practical contribution is reflected in the examination of the reliability of the application of traditional and modern models for predicting company bankruptcy, but also in the creation of own models, which is also a methodological contribution, intended primarily for the Montenegrin market to preventing the occurrence of business failure, and then for all markets of the countries which are at a similar level of economic development. In this way, more efficient and safer business decision-making is directly affected, which is especially important for managers at all levels. It also provides an insight into the direction in which additional knowledge and skills of decision-makers in Montenegro, as a transition country, should be developed in terms of the possibility of applying these models, bearing in mind the specific market requirements. In other words, the business of companies should be supported by advanced knowledge of finance, accounting, and statistics. This is the first research in this scope, according to the knowledge of the author, of the mentioned problem, which will be realized in Montenegro, as a transition country, which is his special practical and theoretical contribution. Hence, the existing literature is given additional value by analysing the concept of company bankruptcy through the application of the most significant models in this area, so this paper gains particular importance and at the same time becomes the basis for directing future research in this area, which will bridge the observed literature gap. The obtained results can serve managers and other decision-makers as guidelines for further improvement of the company’s operations to advance business performance, but also to strengthen the competitiveness of the company, especially if it is taken into account that Montenegro is a transitional country and that the competitiveness of companies with continuous operations is their primary goal. This will also fulfil the requirements of the International Financial Reporting Standards and the International Auditing Standards related to the way of doing business respecting the principle of going concern. In other words, decision-makers must be aware that adequate knowledge and skills, as well as trend monitoring, are prerequisites for the development and better usage of bankruptcy prediction models. Therefore, it is not enough to decide on the application of the model, but to essentially implement them and finally to consider their effects. Therefore, the concept of predicting the bankruptcy of a company should be seen as part of not only business but also corporate strategy, which directly affects the reduction of the number of companies in bankruptcy.

However, it is important to point out that the analysis of this paper implied the prediction of business failure based on financial statements for the year before the onset of bankruptcy. The authors of the research, due to the unavailability of financial reports for the periods before the onset of bankruptcy, did not take into account a wider time frame, which is one of the limitations of the work. In this way, it was not possible to observe the trend of the calculated indicators throughout the period. Another limitation stems from the fact that the research covers only the Montenegrin market, which means that future research can also cover other countries of the Western Balkans, which will provide a clearer insight into the way companies operate in this market, draw general conclusions that can help managers at all levels to detect early signals of business failure.

In terms of methodology, five modern models were included along with the creation of a logit model, so future research can apply the same models in other countries to examine their level of reliability and draw general conclusions based on comparative analysis. Also, future research can include other models for predicting business failure, and test the level of reliability of the created logit models for predicting business failure. And finally, further research on this topic can also include artificial intelligence models, to examine the level of their reliability for application in the Montenegrin market.

## Supporting information

S1 Appendix Logistic regression estimation output. (DOCX)

S1 Data (XLSX)

## References

[1] Ahlstrom D, Arregle J-L, Hitt MA, Qian G, Ma X, Faems D. Managing technological, sociopolitical, and institutional change in the New Normal. J Manag Stud [Internet]. 2020;57(3):411–37. Available from: doi: 10.1111/joms.12569

[2] Pogodina TV, Muzhzhavleva TV, Udaltsova NL. Strategic management of the competitiveness of industrial companies in an unstable economy. J Entrep. Sustain Issu [Internet]. 2020;7(3):1555–64. Available from: doi: 10.9770/jesi.2020.7.3(9).

[3] Arokodare Asikhia. Strategic agility: Achieving superior organizational performance through strategic foresight. Global Journal of Management and Business Research [Internet]. 2020;7–16. Available from: doi: 10.34257/gjmbravol20is3pg7

[4] Jaffari AA, Ghafoor Z. Predicting corporate bankruptcy in Pakistan A comparative study of multiple discriminant analysis (MDA) and logistic regression. Research Journal of Finance and Accounting. 2017;8(3):81–100. ISSN 2222-1697 (Paper) ISSN 2222-2847 (Online).

[5] Nanayakkara KGM, Azeez AA. Predicting corporate financial distress in Sri Lanka: an extension to Z-score model. International Journal of Business and Social Research. 2015;5(03):41–56. Available from: https://thejournalofbusiness.org/index.php/site/issue/view/42.

[6] Fernández-Gámez MÁ, Soria JAC, Santos JAC, Alaminos D. European country heterogeneity in financial distress prediction: An empirical analysis with macroeconomic and regulatory factors. Econ Model [Internet]. 2020;88:398–407. Available from: doi: 10.1016/j.econmod.2019.09.050

[7] Rashid A, Abbas Q. Predicting Bankruptcy in Pakistan. Theoretical & Applied Economics. 2011;18(9).

[8] Rodríguez-Valencia L., Lamothe-Fernández P., & Alaminos D. (2023). The market value of SMEs: a comparative study between private and listed firms in alternative stock markets. Annals of Finance. Available from: doi: 10.1007/s10436-022-00420-z

[9] Alam TM, Shaukat K, Mushtaq M, Ali Y, Khushi M, Luo S, et al. Corporate bankruptcy prediction: An approach towards better corporate world. Comput J [Internet]. 2021;64(11):1731–46. Available from: doi: 10.1093/comjnl/bxaa056

[10] Panigrahi C. M. A. Validity of Altman’s ‘Z’score model in predicting financial distress of pharmaceutical companies. NMIMS journal of economics and public policy, (2019);4(1).

[11] Winarta W, Kuntadi C. Literature review: The effect of company size, company growth, and company liquidity on going concern audit opinion. Dinasti International Journal of Economics, Finance & Accounting [Internet]. 2022;3(4):430–7. Available from: doi: 10.38035/dijefa.v3i4.1438

[12] Begović SV, Tomašević S, Momčilović M. External audit in the function of assuring the correctness of the assumptions about the going concern assumption. BizInfo (Blace) Journal of Economics. 2022;13(1):49–55. Available from: doi: 10.5937/bizinfo2201049V

[13] Abadi K, Purba DM, Fauzia Q. The Impact of Liquidity Ratio, Leverage Ratio, Company Size, and Audit Quality on Going Concern Audit Opinion. Jurnal Akuntansi Trisakti. 2019;6(1):69–82.

[14] Eurostat. Q2 2023: Business bankruptcies at highest level since 2015 [Internet]. Eurostat. 2023 [cited 2024 Mar 7]. Available from: https://ec.europa.eu/eurostat/en/web/products-eurostat-news/w/ddn-20230817-1.

[15] Correa-Mejía DA, Lopera-Castaño M. Financial ratios as a powerful instrument to predict insolvency; a study using boosting algorithms in Colombian firms. Estud Gerenc [Internet]. 2020;229–38. Available from: doi: 10.18046/j.estger.2020.155.3588

[16] Awais M, Hayat F, Mehar N, Hassan W. Do Z-Score and Current Ratio have the Ability to Predict Bankruptcy? Developing Country Studies. 2015;5:30–6. ISSN 2224-607X (Paper) ISSN 2225-0565 (Online).

[17] Sahu PA, Charan P. Ratio analysis is an instrument-For decision making-A study. Asia Pacific Journal of Research. 2013;1(8):36–41. ISSN 2320-5504 (Print).

[18] Anandasayanan S, Subramaniam VA. Predicting Bankruptcy of selected manufacturing companies listed in Colombo Stock Exchange: Applying Altman’S Z-score. 2017; Available from: doi: 10.5281/zenodo.376051

[19] Boshkoska M, Prisagjanec M. Business Success and Failure Prediction Software-BEX Model. Ecoforum Journal. 2017;6(1).

[20] Alaminos D, del Castillo A, Fernández MÁ (2018) Correction: A Global Model for Bankruptcy Prediction. PLOS ONE 13(11): e0208476. Available from: doi: 10.1371/journal.pone.0208476 30485378 PMC6261637

[21] Aminian A, Mousazade H, Khoshkho OI. Investigate the ability of bankruptcy prediction models of Altman and springate and zmijewski and Grover in Tehran stock exchange. Mediterr J Soc Sci [Internet]. 2016; Available from: doi: 10.5901/mjss.2016.v7n4s1p208

[22] Kozjak SK, Sestanj-Peric T, Besvir B. Assessment of Bankruptcy Prediction Models’ applicability In Croatia. In: An Enterprise Odyssey International Conference Proceedings. 2014. ISBN 978-953-6025-91-6.

[23] Manuel Ángel Fernández, José Ramón Sánchez, Alaminos D, Casado G. Predicting going concern opinion for hotel industry using classifiers combination. International journal of scientific management and tourism. 2018 Mar 10;4(1):91–106. ISSN-e 2386–8570, ISSN 2444-0299.

[24] Ahmadi APS, Soleimani B, Vaghfi SH, Salimi M. Corporate bankruptcy prediction using a logit model: Evidence from listed companies of Iran. World Applied Sciences Journal. 2012; 17(9):1143–8.

[25] Alaminos D, Fernández MÁ. Why do football clubs fail financially? A financial distress prediction model for European professional football industry. Gherghina SC, editor. PLOS ONE. 2019 Dec 26;14(12):e0225989 Available from: doi: 10.1371/journal.pone.0225989 31877154 PMC6932787

[26] Alaminos D, Fernández SM, García F, Fernández MA. Data Mining for Municipal Financial Distress Prediction. Lecture notes in computer science. 2018 Jan 1;296–308. Available from: doi: 10.1007/978-3-319-95786-9_23

[27] Alaminos D, Esteban I, Fernández-Gámez MA. Financial Performance Analysis in European Football Clubs. Entropy. 2020; 22(9):1056. Available from: doi: 10.3390/e22091056 33286825 PMC7597129

[28] Panchal MH, Bodar MB, Maurya SR, Tatwadarshi PN. An Innovative Approach to Predict Bankruptcy. VIVA-Tech International Journal for Research and Innovation. 2019;1(2):1–6. ISSN (Online): 2581–7280.

[29] Olson DL, Delen D, Meng Y. Comparative analysis of data mining methods for bankruptcy prediction. Decis Support Syst [Internet]. 2012;52(2):464–73. Available from: doi: 10.1016/j.dss.2011.10.007

[30] Klepáč V, Hampel D. Predicting bankruptcy of manufacturing companies in EU. E+M Ekon Manag [Internet]. 2018; 21(1):159–74. Available from: doi: 10.15240/tul/001/2018-1-011

[31] ‌Alaminos D, Belén Salas M. Tourism Stock Prices, Systemic Risk and Tourism Growth: A Kalman Filter with Prior Update DSGE-VAR Model. Lecture notes in computer science. 2023 Jan 1;167–81. Available from: doi: 10.1007/97

[32] Lakićević M, Mijić K. Pouzdanost primjene modela za predviđanje stečaja preduzeća u Crnoj Gori, XIII Kongres računovođa i revizora Crne Gore—Računovodstvena i revizijska profesija budućnosti: Povjerenja—Integritet—Transparantnost, 2018 (Oktobar 19–21); p. 139–53.

[33] Beaver WH. Financial ratios as predictors of failure. J Acc Res [Internet]. 1966;4:71. Available from: doi: 10.2307/2490171

[34] Altman EI, Iwanicz-Drozdowska M, Laitinen EK, Suvas A. Distressed firm and bankruptcy prediction in an international context: A review and empirical analysis of Altman’s Z-score model. SSRN Electron J [Internet]. 2014; Available from: doi: 10.2139/ssrn.2536340

[35] Seto AA. Altman Z-Score Model, Springate, Grover, Ohlson, and Zmijweski to Assess the Financial Distress Potential of PT. Garuda Indonesia Tbk During and After the Covid-19 Pandemic. Enrichment: Journal of Management. 2022;12(5):3819–26. Available from: doi: 10.35335/enrichment.v12i5.923

[36] S, Sembiring TM. Bankruptcy Prediction Analysis of Manufacturing Companies Listed in Indonesia Stock Exchange. IJEFI. 2015;5(1):354–9.

[37] Ohlson JA. Financial ratios and the probabilistic prediction of bankruptcy. J Acc Res [Internet]. 1980;18(1):109. Available from: doi: 10.2307/2490395

[38] Bateni L, Asghari F. Bankruptcy prediction using logit and genetic algorithm models: A comparative analysis. Comput Econ [Internet]. 2020;55(1):335–48. Available from: doi: 10.1007/s10614-016-9590-3

[39] Viciwati V. Bankruptcy prediction analysis using the Zmijewski model (x-Score) and the Altman model (z-Score). Dinasti International Journal of Economics, Finance & Accounting [Internet]. 2020;1(5):794–806. Available from: doi: 10.38035/dijefa.v1i5.608

[40] Zmijewski ME. Methodological issues related to the estimation of financial distress prediction models. J Acc Res [Internet]. 1984;22:59. Available from: doi: 10.2307/2490859

[41] Machek O. Long-term predictive ability of bankruptcy models in the Czech Republic: Evidence from 2007–2012. Cent Eur Bus Rev [Internet]. 2014;3(2):14–7. Available from: doi: 10.18267/j.cebr.80

[42] Neumaierová I, Neumaier I. INFA Performance Indicator Diagnostic System. Cent Eur Bus Rev [Internet]. 2014;3(1):35–41. Available from: doi: 10.18267/j.cebr.73

[43] Cita M, Stanić M, Stanić Šulentić M. Predicting Bankruptcy of Shipbuilding Companies on the Basic of Financial Statement Data. Journal of Accounting and Management [Internet]. 2019 [accsesed 15.04.2024.]; IX(2):67–76. Avaliable from: https://hrcak.srce.hr/234524.

[44] Belak V, Barać Ž. Business excellence (BEX) indeks-za procjenu poslovne izvrsnosti tvrtki na tržištu kapitala u Republici Hrvatskoj. Računovodstvo, revizija i financije. 2007;17:15–25. ISSN 0353-8087.

[45] Low SW, Nor FM, Yatim P. Predicting corporate financial distress using the logit model: The case of Malaysia. Asian Academy of Management Journal. 2001;6(1):49–61.

[46] Abdullah NAH, Ma’aji MM, Khaw KL-H. The value of governance variables in predicting financial distress among small and medium-sized enterprises in Malaysia. Asian Academy of Management Journal of Accounting & Finance [Internet]. 2016;12(Suppl. 1):75–88. Available from: doi: 10.21315/aamjaf2016.12.s1.4

[47] Pongsatat S, Ramage J, Lawrence H. Bankruptcy prediction for large and small firms in Asia: a comparison of Ohlson and Altman. Journal of Accounting and Corporate Governance. 2004;1(2):1–13.

[48] Alkhatib K, Eqab Al Bzour A. Predicting corporate bankruptcy of Jordanian listed companies: Using Altman and Kida models. Int J Bus Manag [Internet]. 2011;6(3). Available from: doi: 10.5539/ijbm.v6n3p208

[49] Roomi MS, Ahmad W, Ramzan M, Zia-ur-Rehman M. Bankruptcy prediction for non-financial firms of Pakistan. Int J Account Financ Report [Internet]. 2015;5(2):26. Available from: doi: 10.5296/ijafr.v5i2.7782

[50] Niresh J, Pratheepan T. The application of Altman’s z-score model in predicting bankruptcy: Evidence from the trading sector in Sri Lanka. International Journal of Business and Management. 2015;10(12):269–75. Available from: https://ssrn.com/abstract=2698532.

[51] Hanić A, Žunić E, Dželihodžić A. Scoring Models of Bank Credit Policy Management. Economic analysis. 2013;46(1–2):12–27. ID: 198577676.

[52] Ballkoci V, Gremi E. Logit analysis for predicting the bankruptcy of Albanian retail firms. Acad J Interdiscip Stud [Internet]. 2016; Available from: doi: 10.5901/ajis.2016.v5n3s1p137

[53] Smaranda C. Scoring functions and bankruptcy prediction models-case study for Romanian companies. Procedia Economics and Finance. 2014;10:217–26. Available from: doi: 10.1016/S2212-5671(14)00296-2

[54] Makeeva E, Neretina E. The prediction of bankruptcy in a construction industry of Russian Federation. Journal of Modern Accounting and Auditing. 2013;9(2). ISSN 1548-6583.

[55] Celli M. Can Z-score model predict listed companies’ failures in Italy? An empirical test. Int J Bus Manag [Internet]. 2015;10(3). Available from: doi: 10.5539/ijbm.v10n3p57

[56] Pervan I. Predviđanje stečaja—sme proizvodna poduzeća u Hrvatskoj. Zbornik radova Veleučilišta u Šibeniku [Internet]. 2017 [cited 2024 March 08]; 11(3–4):33–45. Available from: https://hrcak.srce.hr/184255.

[57] Zenzerović R. Credit scoring models in estimating the creditworthiness of small and medium and big enterprises. Croatian Operational Research Review [Internet]. 2011 [accessed: 15.04.2024.]; 2(1):143–157. Available from: https://hrcak.srce.hr/96659.

[58] Begović SV, Momčilović M, Tomašević S. Ocena kreditnog boniteta preduzeća Z”-score modelom. 1. Stevan Luković, Milka Grbić. [Internet]. 2014; 52:193–204. Available from: http://www.eknfak.ni.ac.rs/src/Ekonomske-teme.php.

[59] Bešlić Obradović D, Jakšić D, Bešlić Rupić I, Andrić M. Insolvency prediction model of the company: the case of the Republic of Serbia. Econ Res-Ekon Istraž [Internet]. 2018;31(1):139–57. Available from: doi: 10.1080/1331677x.2017.1421990

[60] Liang D, Lu C-C, Tsai C-F, Shih G-A. Financial ratios and corporate governance indicators in bankruptcy prediction: A comprehensive study. Eur J Oper Res [Internet]. 2016;252(2):561–72. Available from: doi: 10.1016/j.ejor.2016.01.012

[61] Shin K-S, Lee Y-J. A genetic algorithm application in bankruptcy prediction modeling. Expert Syst Appl [Internet]. 2002;23(3):321–8. Available from: doi: 10.1016/s0957-4174(02)00051-9

[62] Wu C-H, Fang W-C, Goo Y-J. Variable selection method affects SVM-based models in bankruptcy prediction. In: Proceedings of the 9th Joint International Conference on Information Sciences (JCIS-06). Paris, France: Atlantis Press; 2006. Available from: 10.2991/jcis.2006.114.

[63] IFAC.org. [cited 2024 Mar 7]. Available from: https://www.ifac.org/about-ifac/membership/profile/montenegro.

[64] Gov.me. [cited 2020 June 10]. Available from: http://www.poreskauprava.gov.me/uprava.

[65] Altman EI, Iwanicz-Drozdowska M, Laitinen EK, Suvas A. Financial distress prediction in an international context: A review and empirical analysis of Altman’s Z-score model. Journal of International Financial Management & Accounting. 2017;28(2):131–71. Available from: doi: 10.1111/jifm.12053

[66] Agarwal A, Patni I. Bankruptcy prediction models: An empirical comparison. International Journal of Innovative Technology and Exploring Engineering. 2019;8(6):131–9.

[67] Bărbuță-Mișu N, Madaleno M. Assessment of bankruptcy risk of large companies: European countries evolution analysis. J Risk Fin Manag [Internet]. 2020;13(3):58. Available from: doi: 10.3390/jrfm13030058

[68] Prasetiyani E, Sofyan M. Bankruptcy analysis using Altman Z-score model and Springate model in retail trading company listed in Indonesia Stock Exchange. Ilomata International Journal of Tax and Accounting. 2020;1(3):139–44. Available from: doi: 10.52728/ijtc.v1i3.98

[69] Muzani M, Yuliana I. Comparative analysis of Altman, Springate and Zmijewski models in predicting the bankruptcy of retail companies in Indonesia and Singapore. TIJAB (The International Journal of Applied Business). 2021;5(1):81–93. Available from: 10.20473/tijab.V5.I1.2021.81-93.

[70] Schönfeld J, Kuděj M, Smrčka L. Financial health of enterprises introducing safeguard procedure based on bankruptcy models. J Bus Econ Manage [Internet]. 2018;19(5):692–705. Available from: doi: 10.3846/jbem.2018.7063

[71] Onofrei M, Lupu D. The Modeling Of Forecasting The Bankruptcy Risk In Romania. Romania Economic Computation & Economic Cybernetics Studies & Research. 2014;48(3).

[72] Heaton JB. The Altman Z score does not predict bankruptcy. SSRN Electron J [Internet]. 2020; Available from: doi: 10.2139/ssrn.3570149

[73] World economic forum [Internet]. Weforum.org. [cited 2020 Aug 5]. Available from: http://www3.weforum.org/docs/WEF_TheGlobalCompetitivenessReport2019.pdf.

[74] Wahyuningsih T, Venusita L. Financial Analysis Of Retail Companies Using The Altman, Springate, Zmijewski, Fulmer, And Grover Bankruptcy Prediction Models: Case Study of Retail Companies Listed on the Indonesia Stock Exchange for the Period 2019–2020. Journal Of Accounting, Entrepreneurship and Financial Technology (Jaef). 2022;3(2):149–68. Available from: doi: 10.37715/jaef.v3i2.2661

[75] Salkic A. Testing possibility of establishing creditworthiness of small and medium enterprises in Bosnia and Herzegovina by applying Kralicek DF indicator. Economic Review: Journal of Economics and Business. 2013;11(2):57–70. Available from: https://hdl.handle.net/10419/193829.

[76] Mijić K. Implementation of Business Performance Projection Models Based on Data from Financial Statements. Financing-naučni časopis za ekonomiju. 2020;11:3–15. Available from: doi: 10.7251/FIN2003003M

[77] Ptak-Chmielewska A. Bankruptcy prediction of small-and medium-sized enterprises in Poland based on the LDA and SVM methods. Statistics in Transition New Series. 2021;22(1):179–95. Available from: doi: 10.21307/stattrans-2021-010

[78] Ogachi D, Ndege R, Gaturu P, Zoltan Z. Corporate bankruptcy prediction model, a special focus on listed companies in Kenya. J Risk Fin Manag [Internet]. 2020;13(3):47. Available from: doi: 10.3390/jrfm13030047

[79] Alaminos D, Guillén-Pujadas M, Emili Vizuete-Luciano, José María Merigó. What is going on with studies on financial speculation? Evidence from a bibliometric analysis. International review of economics & finance. 2024 Jan 1; 89:429–45. Available from: doi: 10.1016/j.iref.2023.10.040

