# Supplementary material for: Modern models for predicting bankruptcy to detect early signals of business failure: Evidence from Montenegro
Source: PLoS One. 2024 May 21;19(5):e0303793. doi: 10.1371/journal.pone.0303793 (PMC11108218; doi:10.1371/journal.pone.0303793)
Supplement: S1 Appendix — (DOCX) [file pone.0303793.s001.docx]

# **APPENDIX**

**Table A1. Likelihood Ratio Tests**

| **Likelihood Ratio Tests** | | | | |
| --- | --- | --- | --- | --- |
| **Effect** | Model Fitting Criteria | Likelihood Ratio Tests | | |
|  | -2 Log Likelihood of Reduced Model | Chi-Square | df | Sig. |
| **Intercept** | 67.455 | 13.195 | 1 | <.001 |
| **DI** | 90.356 | 36.096 | 1 | <.001 |
| **TR** | 83.298 | 29.038 | 1 | <.001 |
| **EBIT** | 54.908 | .648 | 1 | .421 |
| **Current liquidity** | 57.045 | 2.786 | 1 | .095 |
| **ROA** | 55.047 | .787 | 1 | .375 |
| **ROE** | 54.461 | .201 | 1 | .654 |
| The chi-square statistic is the difference in -2 log-likelihoods between the final model and a reduced model. The reduced model is formed by omitting an effect from the final model. The null hypothesis is that all parameters of that effect are 0. | | | | |

Source: authors' processing

**Table A2. Parameter Estimates**

| Parameter Estimates | | | | | | | | | |
| --- | --- | --- | --- | --- | --- | --- | --- | --- | --- |
| Bankruptcy ^a^ | | B | Std. Error | Wald | df | Sig. | Exp(B) | 95% Confidence Interval for Exp(B) | |
|  |  |  |  |  |  |  |  | Lower Bound | Upper Bound |
| .00 | Intercept | 2.427 | .776 | 9.781 | 1 | .002 |  |  |  |
|  | DI | -.057 | .015 | 14.943 | 1 | <.001 | .945 | .918 | .972 |
|  | TR | 2.975 | .901 | 10.903 | 1 | <.001 | 19.585 | 3.350 | 114.490 |
|  | EBIT | .000 | .000 | .539 | 1 | .463 | 1.000 | 1.000 | 1.000 |
|  | Current liquidity | -.086 | .065 | 1.747 | 1 | .186 | .918 | .809 | 1.042 |
|  | ROA | .039 | .044 | .798 | 1 | .372 | 1.040 | .954 | 1.134 |
|  | ROE | .002 | .004 | .252 | 1 | .616 | 1.002 | .994 | 1.010 |
| a. The reference category is non-bankruptcy. | | | | | | | | | |

Source: authors' processing
